# Protein‐Derived Signal Peptides Induced by *Agrobacterium* Infection Promote the Secretion of Recombinant Proteins in *Nicotiana benthamiana*

**DOI:** 10.1111/pbi.70498

**Published:** 2025-12-16

**Authors:** Hiroyuki Kajiura, Kana Yamamoto, Ryo Misaki, Kazuhito Fujiyama

**Affiliations:** ^1^ International Center for Biotechnology The University of Osaka Osaka Japan; ^2^ Institute for Open and Transdisciplinary Research Initiatives (OTRI), The University of Osaka Osaka Japan; ^3^ Department of Biotechnology, Graduate School of Engineering The University of Osaka Osaka Japan; ^4^ Osaka University Cooperative Research Station in Southeast Asia (OU:CRS), Faculty of Science, Mahidol University Bangkok Thailand

**Keywords:** apoplast, *Nicotiana benthamiana*, pathogen‐related protein, recombinant protein secretion, signal peptide

## Abstract

Plants are promising next‐generation hosts for recombinant protein production; however, major challenges remain with regard to enhancing the efficiency of downstream processing, particularly in the removal of cellular residues and purification of the expressed proteins. Strategies to overcome these limitations include targeting expressed recombinant proteins within a specific organelle or directing their secretion into the extracellular space, thereby facilitating purification by collecting the target matrix. In this study, we focused on protein secretion mechanisms and identified two pathogenesis‐related proteins, glucan endo‐1,3‐β‐glucosidase (GN) and chitinase 8 (Chi8), which accumulated in the apoplast washing fluid following *Agrobacterium* infiltration of *Nicotiana benthamiana* leaves. Both proteins contained signal peptides (SPs), SP_GN_ and SP_Chi8_, respectively. Although the intracellular accumulation of GFP was comparable regardless of the expression level, fusion with either SP_GN_ or SP_Chi8_ resulted in GFP accumulation within the apoplast. In contrast, in *N. benthamiana*, a mammalian‐derived SP was less effective in facilitating GFP secretion than the plant‐derived SPs. Additionally, replacing the SP of the mammalian‐derived protein β‐glucocerebrosidase (GCase) with SP_GN_ or SP_Chi8_ enhanced the secretion of GCase into the apoplast, indicating their applicability in protein production. Moreover, SP_GN_ and SP_Chi8_ directed the expressed proteins into the culture medium of *N. benthamiana* suspension cells. These results indicate that SP_GN_ and SP_Chi8_ function as effective secretion signals and highlight the potential application of endogenous SPs for enhancing recombinant protein production in plants.

## 1 Introduction

Recently, plants have gained increasing attention as platforms for the production of recombinant proteins, specifically biopharmaceuticals. They enable large‐scale, pathogen‐free, and cost‐effective production while allowing posttranslational modifications (Buyel 2019; Paul and Ma 2011). Additionally, key technological innovations have made plant‐based expression systems more competitive. These include vacuum infiltration of *Agrobacterium* into intact leaves (agroinfiltration) (Kapila et al. 1997), the use of virus‐based vectors, and the magnifection approach, which combines the advantages of both methods (Marillonnet et al. 2005). As a result, recombinant protein production in plants now rivals that in other heterologous hosts, such as bacteria, yeast, insect cells, and mammalian cells. These advances have facilitated molecular farming, defined as the use of plants or plant systems, such as suspension‐cultured cells (Karki et al. 2021) and hairy root cultures (Skarjinskaia et al. 2013), to produce recombinant proteins, including biopharmaceuticals. The first plant‐made pharmaceutical, Elelyso, a therapeutic for Type I Gaucher disease produced in suspension‐cultured carrot cells, was approved by the U.S. Food and Drug Administration (FDA) in 2012 (Fox 2012; Mor 2015). Since then, over 35 plant‐produced biopharmaceuticals have undergone clinical trials (Sethi et al. 2021), and there are numerous additional examples of the potential for recombinant protein production in plants and plant systems. Currently, the focus of molecular farming is the optimisation of total production efficiency, including post‐translational modifications and/or downstream processing, such as material recovery, protein extraction, and purification steps. Consequently, it would also be desirable to establish strategies that enhance recombinant protein production by focusing on downstream processes.

In eukaryotic systems, N‐terminal signal peptides (SPs), cellular targeting signals, and/or C‐terminal retention signals direct the final product to specific organelles. SPs, consisting of short hydrophobic peptides, play an essential role in the translocation of cytosolic ribosome‐translated nascent polypeptides into the endoplasmic reticulum (ER). They function by interacting with the signal recognition particles (SRPs), translocon Sec61, and signal peptidase complex (Liaci and Förster 2021; Owji et al. 2018), thereby influencing the efficiency of protein secretion and post‐targeting functions. C‐terminal retention signals facilitate ER targeting and accumulation, whereas SPs mediate secretion, thus enabling the purification and production of more homogeneous recombinant proteins via secretory pathways. For example, targeting recombinant proteins to the ER by fusing them with C‐terminal KEDL/HDEL or SEKDEL sequences has been found to enhance recombinant protein productivity because of the involvement of less active proteases (Petruccelli et al. 2006; Schouten et al. 1996). In addition, vacuole targeting of recombinant proteins is effective for *N*‐glycoproteins, such as β‐glucocerebrosidase (GCase), a glucosylceramidase that degrades glucocerebroside to yield glucose and ceramide in mammalian cells (Boer et al. 2020), and is commercially available as Elelyso. Because GCase requires mannose residue(s) at the non‐reducing terminal for endogenous activity (He et al. 2014; Shaaltiel et al. 2007), targeting GCase to the vacuole, where glycan‐modifying enzymes are localised, can be considered an ideal strategy. Active type I collagen, immunoglobulin, α1‐proteinase inhibitor, and interleukin‐6 have also been successfully produced via their accumulation in vacuoles (Marin Viegas et al. 2017; Ocampo et al. 2016; Stein et al. 2009). Numerous proteins, such as cytokines and antibodies, are produced in their mature forms via translocation into the apoplasts (Soleimanizadeh et al. 2022; Teh and Kavanagh 2010; Wilbers et al. 2016; Wirth et al. 2004), the extracellular spaces in plants where secreted proteins accumulate, contributing to nutrient and water transport, as well as facilitating the synthesis of molecules involved in plant stress‐defence responses (Dora et al. 2022; Sattelmacher 2001). The induction of protein secretion into the apoplast enables the production of mature proteins, as they undergo processing during passage through the ER and Golgi apparatus. Accordingly, a secretory system is required to produce mature and active proteins in plants. In suspension‐cultured cells, SPs facilitate the secretion of several active biopharmaceutical proteins (Karki et al. 2021; Xu et al. 2011), and translocation into the apoplast or secretion of the target recombinant protein into the medium can serve as effective strategies for producing and accumulating high‐quality biopharmaceutical proteins.

Recently, plant molecular farming involving agroinfiltration and transient expression in *Nicotiana benthamiana* has become an essential technique for recombinant protein production. It can be speculated that *Agrobacterium* infection‐induced mRNAs and/or proteins may contain factors that enhance the production of recombinant protein. Specifically, proteins induced by the plant immune system against exogenous pathogenic bacterial infections may accumulate in the apoplast. Indeed, the heightened secretion of pathogenesis‐related (PR) proteins, such as chitinases and β1,3‐glucanases (Sels et al. 2008; Sinha et al. 2014), into the apoplast has been observed following agroinfiltration (Goulet et al. 2010). Given the necessity of active SPs for the recruitment of proteins to the apoplast, SPs from endogenous apoplastic proteins induced by agroinfiltration are considered applicable for recombinant protein secretion. SPs derived from apoplast‐localised PR proteins have been established to have secretory capacities and are used for protein secretion (Dagvadorj and Solomon 2021; Debler et al. 2021; Wilbers et al. 2016). Notably, however, whereas recombinant protein production using mammalian‐derived SPs may induce leaf necrosis, resulting in inadequate protein production, plant‐derived SPs can alleviate these effects (Gils et al. 2005; Wilbers et al. 2016). These findings thus indicate that plant‐derived SPs can contribute to enhancing the efficiency of recombinant protein production in *N. benthamiana* for transient expression. Similarly, *N. benthamiana*‐derived SPs may be of particular efficacy in this regard for agroinfiltration‐mediated transient expression.

In this study, we focused on isolating SPs that can enhance transient protein production in *N. benthamiana* and thereby facilitate protein translocation/secretion for subsequent purification. We identified two apoplast‐derived *N. benthamiana* proteins that were strongly induced in response to agroinfiltration. The effects of SPs from these proteins on protein secretion into the apoplast were evaluated by fusing each SP with either green fluorescent protein (GFP) or the model *N*‐glycoprotein GCase, and we subsequently analysed protein secretion, activity, and *N*‐glycosylation. In addition, the versatility and applications of these SPs were investigated in *N. benthamiana* suspension‐cultured cells using constructs comprising GFP with SPs. The characteristics of the two novel SPs and their potential for recombinant protein production are discussed, along with the identification of novel and functional factors for the production and secretion of recombinant proteins.

## 2 Results

### 2.1 *Agrobacterium* Infection Promotes the Accumulation of Two Proteins, Glucan Endo‐1,3‐β‐Glucosidase and Chitinase 8, in Apoplast Washing Fluid

**FIGURE 1:**
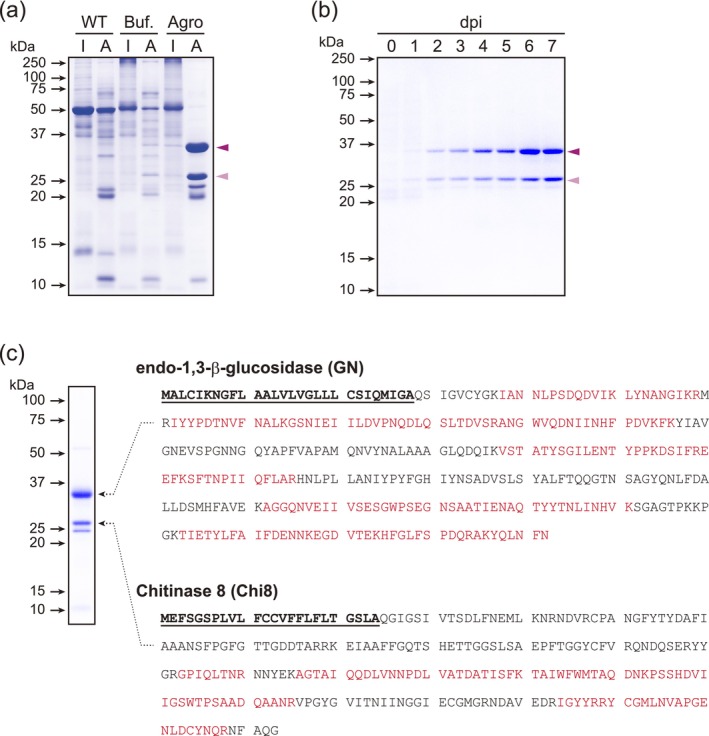
Identification of AWF proteins induced by agroinfiltration. (a) CBB staining of total intracellular and AWF proteins. Total proteins from intracellular and apoplast samples obtained from non‐infected, buffer‐infiltrated, and agroinfiltrated leaves were separated by SDS‐PAGE and visualised using CBB staining. The two predominant proteins, indicated by triangles, were detected as agroinfiltration‐AWF‐specific proteins. WT, extracts from wild‐type leaves; Buf., extracts from buffer‐infiltrated leaves; Agro, extracts from leaves infiltrated with *Agrobacterium* harbouring the pRI201 vector. I and A indicate intracellular and AWF, respectively. (b) Time‐course analysis of the induction of two predominant agroinfiltrated‐AWF‐specific proteins. Total proteins in the AWF of agroinfiltrated leaves at 0–7 dpi were analysed. Triangles indicate agroinfiltrated‐AWF‐specific proteins. (c) Identification of the two predominant proteins, GN and Chi8. The CBB‐stained band was excised, digested with trypsin, analysed by nanoLC‐MS/MS, and identified by a MASCOT search and a subsequent BLAST search using the Sol Genomic Network database. The amino acid sequences identified by both database searches are indicated in red, whereas those not identified are indicated in black. Bold and underlined amino acids indicate the putative signal sequences.

To identify novel SPs for protein secretion, we focused on leaf proteins induced by agroinfiltration that accumulate in the apoplast washing fluid (AWF). Leaves from wild type, buffer‐infiltrated, and *Agrobacterium*‐infiltrated samples were separated into intracellular and AWF proteins, and we compared the proteins in each fraction (Figure 1a). In line with expectations, *Agrobacterium* infection induced the accumulation of two major proteins. Notably, in response to buffer infiltration, we detected virtually no accumulation of these proteins in either the AWF or intracellularly. Time‐course analysis revealed that these two proteins accumulated in a time‐dependent manner from 2 to 5 days post‐infiltration (dpi), reaching saturation at 6 dpi (Figure 1b). These results thus indicate that these two proteins were induced by agroinfiltration, but not by buffer infiltration, and that they were secreted into the apoplast.

To identify the proteins, in‐gel digested peptides were analysed using nanoflow liquid chromatography–tandem mass spectrometry (nanoLC–MS/MS) analysis, followed by a Mascot search. Using this strategy, we identified two proteins in *Nicotiana tabacum* . To confirm their homologues in *N. benthamiana*, a BLAST search of the Sol Genomic Network database was performed using the two proteins as queries, and two pathogenic proteins, namely, endo‐1,3‐β‐glucosidase (GN) and chitinase 8 (Chi8), were identified (Figure 1c and Figure S1). Mascot analysis revealed that the peptides matching *N. tabacum* GN and Chi8 were identical to those of *N*. *benthamiana* GN and Chi8, respectively (Figure S1).

GN and Chi8 are classified as Glycoside Hydrolase Family 17 and 19, respectively, in the Carbohydrate‐Active EnZymes (CAZy) database (http://www.cazy.org/) and are members of the PR protein family, categorised as PR‐2 and PR‐3, respectively (Li et al. 2018; Van Loon et al. 2006). In *N. benthamiana*, the GN and Chi8 proteins comprise 342 (37.7 kDa) and 253 (27.7 kDa) amino acids, respectively, with amino acid sequence similarities between *N. benthamiana* and *N. tabacum* being 90.9% and 96.0%, respectively (Figure S1). AlphaFold predictions revealed that, compared with their catalytic domains, both GN and Chi8 possess more flexible and hydrophobic N‐terminal regions. These regions, comprising 28 and 24 amino acids in GN and Chi8, respectively, were predicted to form α‐helices. This α‐helix structure is an essential feature of N‐terminal SPs; specifically, the H‐region that is generally conserved for recognition by SRPs (Figure S2). These regions, with molecular masses of 2.9 and 2.6 kDa, respectively, met the criteria for SPs. Consequently, the calculated masses of GN and Chi8 without their SPs (34.8 and 25.1 kDa, respectively) corresponded to the predominant proteins induced by agroinfiltration. In addition, the results of ApoplastP prediction (https://apoplastp.csiro.au/) indicated that GN and Chi8 localise in the apoplast, with probability scores of 0.65 and 0.82, respectively (proteins with a probability > 0.55 are classified as apoplastic proteins (Sperschneider et al. 2018)). These findings thus provide evidence that the agroinfiltration‐induced GN and Chi8 proteins accumulated in the AWF lack SPs. However, the presence of potential SP regions indicates that these sequences can be used to mediate recombinant proteins into the apoplast.

### 2.2 SP_GN_ and SP_Chi8_ Effectively Guide Expressed GFP Into the Apoplast

**FIGURE 2:**
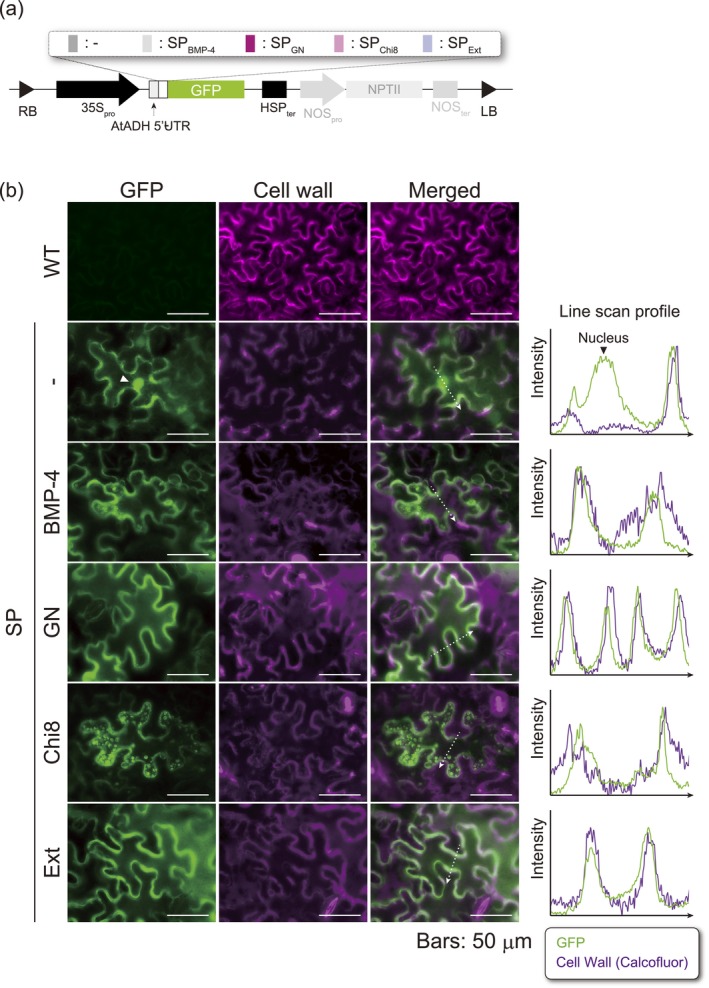
Subcellular localisation analysis of GFP with a novel signal peptide. (a) Schematic representation of the expression vectors of GFP with a signal peptide. CaMV35S_PRO_, cauliflower mosaic virus 35S promoter; HSP_Ter_, heat shock protein terminator from Arabidopsis; AtADH, 5′ untranslated region of Arabidopsis alcohol dehydrogenase gene. The backbone of the expression vector is pRI201‐AN. (b) Microscopic analysis of *N. benthamiana* leaf epidermis cells at 5 dpi. (Left) Dual‐colour imaging by fluorescent microscopy of a transiently GFP‐expressing leaf. Left panel, GFP; middle panel, cell wall staining with calcofluor; right panel, merged images of the two fluorescence signals. The white dotted arrows indicate the scanned line for the line scan profile of each fluorescence intensity. White triangles indicate GFP signals localised in the nucleus in samples without a signal peptide. Bars: 50 μm. (Right) Fluorescence intensity line scan profile generated along the white dotted arrow shown in the merged figures. Green, relative signal intensity of GFP; purple, relative signal intensity of the cell wall stained with calcofluor.

To assess whether specific regions function as SPs for the secretion of proteins into the apoplast, we transiently expressed GFP fusion proteins carrying the putative N‐terminal SPs of GN (SP_GN_) or Chi8 (SP_Chi8_) (Figure 2a) in *N. benthamiana* leaves, and analysed GFP localisation microscopically (Figure 2b). GFP is widely used to examine protein secretion in plants (Larson 2017). As a control, GFP was fused with SPs from human bone morphogenetic protein 4 (BMP‐4; SP_BMP‐4_) and *N. benthamiana* extensin (SP_Ext_), derived from mammalian and plant sources, respectively. Recombinant BMP‐4 has been found to be secreted into the medium when expressed in CHO cells (Kim et al. 2016), and hence SP_BMP‐4_ can be used as a control to evaluate whether an SP that functions in mammalian cells is also functional in plants. In addition, given that the functionality of SP_Ext_ in *N. benthamiana* has been reported (Jiang et al. 2020), we used it as a positive control for assessing secretion. The SPs used in this study are listed in Table 1. We accordingly found that, consistent with apoplastic secretion, all GFPs fused with plant‐derived SPs (SP_GN_, SP_Chi8_, and SP_Ext_) localised primarily around the cell wall. In contrast, the distribution of GFP lacking an SP was characterised by both apoplastic and nuclear localisations (white triangles in Figure 2b and Figure S3). These results reveal that plant‐derived SPs are required for the translocation/secretion of the target protein in *N. benthamiana*, whereas mammalian‐derived SP_BMP‐4_ is non‐functional.

**TABLE 1:** Signal peptide sequences used in this study.

Protein	Signal peptide	Length (aa)	Ref.
GN	MALCIKNGFL AALVLVGLLL CSIQMIGA	28	
Chi8	MEFSGSPLVL FCCVFFLFLT GSLA	24	
Extensin	MGKMASLFAT LLVVLVSLSL ASESSA	26	Jiang et al. (2020)
BMP‐4^a^	MIPGNRMLMV VLLCQVLLG	19	
GCase^b^	MEFSSPSREE CPKPLSRVSI MAGSLTGLLL LQAVSWASG	39	

^a^ The sequence is predicted by SignalP (https://services.healthtech.dtu.dk/services/SignalP‐5.0/).

^b^ The sequence is from the entry of P04062 in UniProt (https://www.uniprot.org/).

**FIGURE 3:**
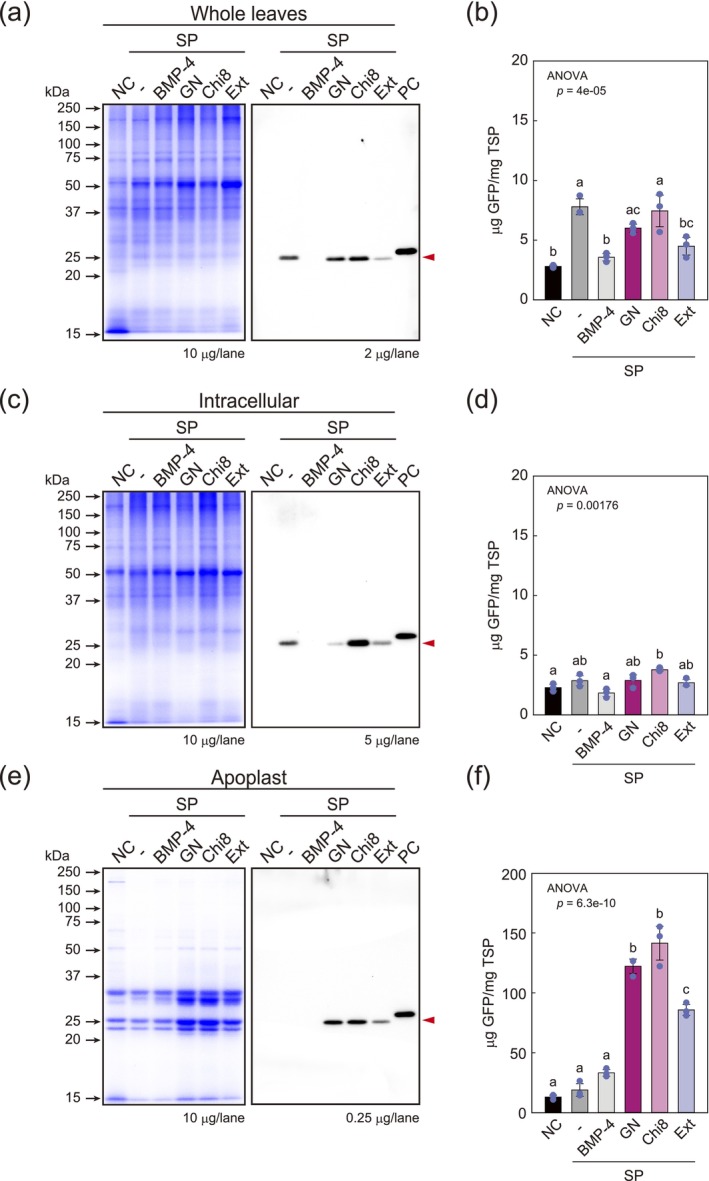
Evaluation of GFP production levels in intracellular compartments and AWF from transiently expressed leaves. CBB staining and Western blotting analysis of GFP in (a) whole leaves, (c) intracellular proteins, and (e) AWF proteins. Total proteins were extracted from whole leaves, leaves after AWF preparation as an intracellular fraction, and AWF itself. GFPs produced in each sample were then detected using an anti‐GFP antibody. Red triangles indicate the GFP produced in each sample. (b, d, f) GFP amounts produced in (b) whole leaves, (d) intracellular protein, and (f) AWF protein. NC, negative control, vector expression; −, Without a signal peptide. Production was calculated as μg/mg TSP. Data are the averages of biological replicates (*n* = 3). Error bars represent the standard deviation of the mean, and three replicates are shown as dots. A one‐way analysis of variance (ANOVA) was performed to compare the means between the groups. The results showed a significant difference among the groups (*p* < 0.05). The *p*‐value for ANOVA is shown in the Figure. Tukey's HSD post hoc test was conducted for pairwise comparisons of the results.

Subsequently, we measured the accumulation of transiently expressed GFP in the AWF and found that there was no significant difference in GFP production in whole leaves or intracellular GFP, with or without an SP (Figure 3a–d). Given that the levels of GFP expression with or without an SP were similar (Figure S4), indicating that SPs have little or no effect on enhancing the production of exogenous protein. In contrast, we detected a significant increase in the amount of GFP in AWF when this protein was fused to plant‐derived SPs (Figure 3e). Secretion ratios were higher in the presence of SPs than in their absence, with SP_GN_ and SP_Chi8_ being more effective than SP_Ext_ (Table 2). Compared with the absence of an SP, the presence of SP_GN_ and SP_Chi8_ enhanced the secretion of GFP by approximately 6.4‐ and 7.5‐fold, respectively, which was more significant than that observed for SP_Ext_ (showing a 4.5‐fold increase in fluorescence) (Figure 3f). The secreted GFP mediated by SP_GN_ and SP_Chi8_ accounted for approximately 12.2% and 14.1% of the total protein in the AWF, respectively (Table 2). Comparatively, although the amount differed depending on the condition of the infected leaves and samples, approximately 13–22 μg/g fresh weight (FW) of GFP was produced intracellularly, accounting for approximately 0.3%–0.4% of the total soluble protein. These results thus indicate that plant‐derived SPs enhance the accumulation of target proteins in the apoplast, with SP_GN_ and SP_Chi8_ being more effective in guiding GFP production into the apoplast.

**TABLE 2:** The productivity of GFP in whole, intracellular, and AWF and the secretion efficiencies.

	Whole		Intracellular		AFW		Secretion ratio (%)
	% TSP	μg GFP/g FW	% TSP	μg GFP/g FW	% TSP	μg GFP/g FW	
—	0.79	38.7	0.29	16.8	1.90	1.4	7.7
SP_GN_	0.60	39.2	0.29	15.2	12.24	15.1	49.8
SP_Chi8_	0.74	48.7	0.38	22.2	14.17	20.8	48.4
SP_Ext_	0.49	28.8	0.27	13.7	8.59	9.4	40.7

*Note:* The secretion ratio is calculated from the following formula; each μg GFP/g FW of AWF/(Intracellular + AWF). Abbreviation: TSP, Total soluble protein.

### 2.3 SP_GN_ and SP_Chi8_ Positively Contribute to GCase Accumulation in the Apoplast Washing Fluid

**FIGURE 4:**
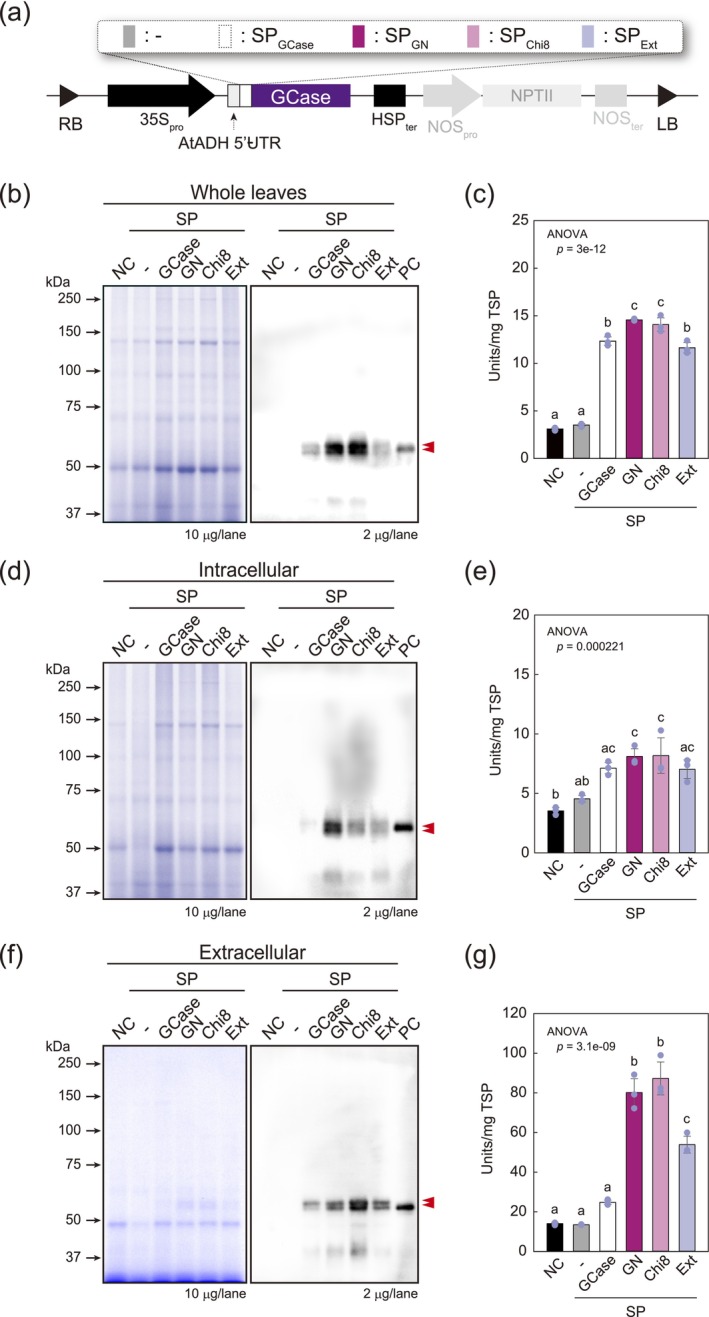
Production of GCase as a model protein. (a) Schematic representation of the expression vectors of GCase with a signal peptide. The abbreviations are the same as those used in Figure 2a. CBB staining and Western blotting analysis of GCase in (b) whole leaves, (d) intracellular protein, and (f) AWF protein. Red triangles indicate GCase produced in each sample. NC: Negative control, vector expression; −: Without signal peptide. The samples were prepared as described in Figure 3. The GCases produced in each sample were detected using an anti‐GCase antibody. Red triangles indicate the GCase produced in each sample. (c, e, g) GCase amounts produced in (c) whole leaves, (e) intracellular protein, and (g) AWF protein. Ten and 5 μg of total proteins were loaded for CBB and Western blotting analyses, respectively. NC, Negative control, vector expression; −, Without signal peptide. The production was calculated as units/mg TSP. Data are averages of biological replicates (*n* = 3). Error bars represent the standard deviation of the mean, and three replicates are shown as dots. Statistical analyses, including ANOVA and post hoc analysis using Tukey's HSD test, were performed as described in Figure 3.

Next, we examined the versatility of SP_GN_ and SP_Chi8_ using the model protein GCase. In the native GCase, an SP with an α‐helical structure functions as a secretory signal for lysosome targeting (Table 1). GCase is secreted into the AWF using its inherent SP. Consequently, to more accurately evaluate the secretory efficiency of SP_GN_ and SP_Chi8_ in *N. benthamiana*, it is preferable to use the SP of GCase (SP_GC_) rather than SP_BMP‐4_. To confirm this hypothesis, we constructed GCase expression vectors harbouring SPs (Figure 4a), which were transiently expressed in *N. benthamiana*. As anticipated, the expression of GCase in *N. benthamiana* resulted in the accumulation of GCase, both intracellularly and in the AWF (Figure 4b–g, Table 3). Notably, whereas the presence of the native SP facilitated the secretion of 2.0 units/g FW GCase, following SP substitution, we detected the secretion of 10–20 units/g FW of this protein, thereby indicating that substituting the native SP of GCase with a plant‐derived SP contributed to an increase in the secretion of approximately 5‐fold per gram of leaf. Interestingly, the accumulation of GCase in the absence of any SP was lower, which can probably be attributed to the loss of an essential factor for correct localisation. Except for GCase lacking an SP, the levels of GCase activity in the whole leaves were similar, indicating that SPs do not contribute to any substantial enhancement of GCase production.

**TABLE 3:** The activity of GCase in whole, intracellular, and AWF and the secretion efficiencies.

	Unit/g FW			Secretion ratio (%)
	Whole	Intracellular	AWF	
SP_GCase_	92.1	9.9	2.2	18.2
SP_GN_	190.7	45.8	21.3	31.7
SP_Chi8_	173.6	55.2	19.7	26.3
SP_Ext_	93.3	35.1	10.8	23.5

*Note:* The secretion ratio is calculated from the following formula: each Unit/g FW of AWF/(Intracellular + AWF).

**FIGURE 5:**
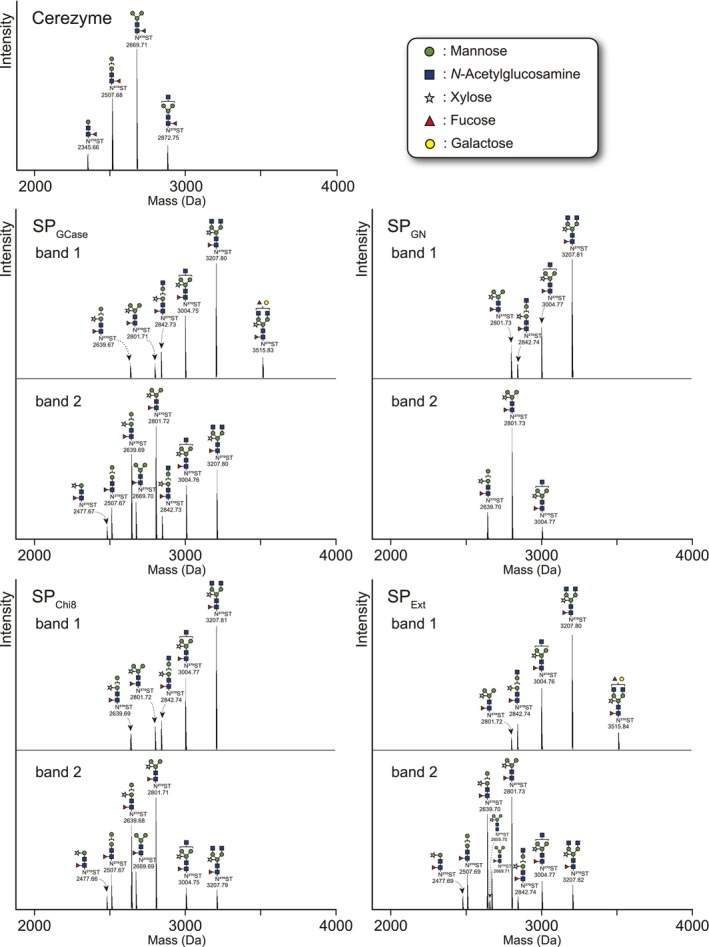
*N*‐Glycosylation analysis of Asn270 in mature GCase. Mammalian‐derived GCase, Cerezyme, and upper GCase (band 1) and lower GCase (band 2) were excised from the CBB‐stained gel, followed by in‐gel trypsin digestion and nanoLC‐MS/MS analysis. All signals of *m/z* corresponding to *N*‐glycopeptide and the *N*‐glycan structures are shown. The symbols used for the *N*‐glycan structures are shown in the small window. The ratios of *N*‐glycans and other *N*‐glycosylation are shown in Table S2 and Figure S5, respectively.

It should be noted that on analysis, all SP‐free GCases showed two bands (Figure 4b,d,f). In‐gel digestion followed by nanoLC‐MS/MS analysis revealed that the two GCase forms were characterised by differences in their *N*‐glycan structures. GCase has five potential *N*‐glycosylation sites, four of which are *N*‐glycosylated (Brumshtein et al. 2006). *N*‐Glycopeptide analysis revealed that GCase with a higher molecular mass was *N*‐glycosylated, predominantly with *N*‐acetylglucosamine (GlcNAc) residues at the non‐reducing terminus. In contrast, the GCase with a lower molecular mass was *N*‐glycosylated with plant‐specific and typical *N*‐glycans without GlcNAc residues (Figure 5, Figure S5, and Table S1). Consequently, the two GCase forms expressed in agroinfiltrated leaves differed with respect to the presence or absence of GlcNAc residues on the non‐reducing termini of *N*‐glycans. Notably, plant‐derived SPs facilitated GCase translocation/secretion, resulting in its accumulation in the AWF as a CBB‐visible band, while retaining its enzymatic activity (Figure S6). These results indicate that SP_GN_ and SP_Chi8_ can effectively mediate the secretion of functional GCases in *N. benthamiana*.

### 2.4 SP_GN_ and SP_Chi8_ Can Be Used for Target Protein Secretion in Suspension‐Cultured Cells

**FIGURE 6:**
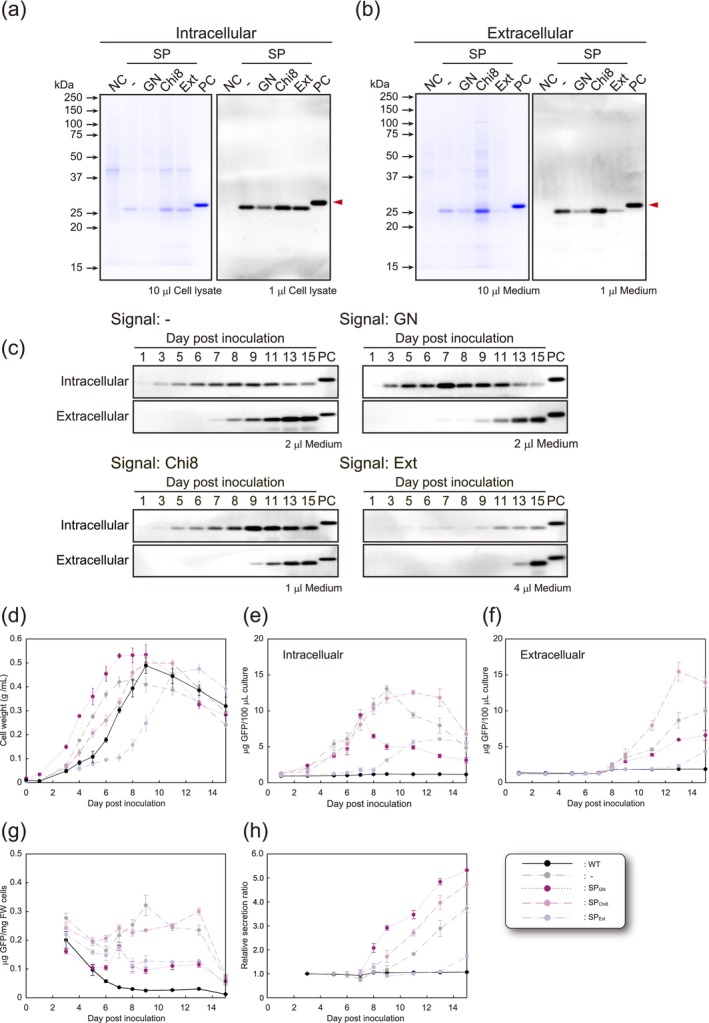
GFP production in suspension‐cultured cells. CBB and Western blotting analysis of GFP in (a) the intracellular fraction and (b) the extracellular fraction of 15‐day‐old *N. benthamiana* suspension‐cultured cells. Red triangles indicate GFP produced in each sample. (c) Time‐course analysis of GFP accumulation and secretion was conducted in both the intracellular and extracellular fractions, respectively. The numbers at the top represent the number of days after subculture. PC: Positive control for recombinant GFP. Each change in (d) cell growth, (e) amount of GFP produced and accumulated intracellularly, (f) amount of GFP secreted into the medium, (g) GFP ratio per FW cells, and (h) relative secretion rates is shown. Error bars represent the standard deviation of the mean.

To assess the functionality of SPs in cultured cells, constructs, excluding SP_BMP‐4_ (Figure 2a), were used to generate *N. benthamiana* suspension‐cultured cells expressing GFP. High‐fluorescence calli were used to establish suspension cultures, all of which produced GFP both intra‐ and extracellularly (Figure 6a,b). GFP lacking an SP was secreted into the culture medium. The fluorescence intensity of GFP in each medium gradually increased after subculture, reaching peaks on days 8, 7, 9, and 13 in the No‐SP, SP_GN_, SP_Chi8_, and SP_Ext_ cultures, respectively (Figure 6c). Although growth rates differed (Figure 6d), transgene and GFP secretion appeared to have minimal effects on cell growth. Analysis of intracellular and secreted GFP revealed differences between intracellular production levels and those of secreted protein in the medium (Figure 6e,f). The ratio of GFP to total intracellular protein content remained independent of the cultivation duration and was maintained at almost constant levels between days 5 and 13 (Figure 6g). These results indicate that increasing the number of cells in the culture facilitated the secretion of larger amounts of GFP. Interestingly, we found that in transient expression in *N. benthamiana* plants, SP_Chi8_ functioned as a more effective SP than SP_GN_, whereas, conversely, SP_GN_ was more effective than SP_Chi8_ in suspension‐cultured cells (Figure 6h). Compared with cells lacking an SP (No‐SP), SP_GN_ and SP_Chi8_ contributed to the production of significantly higher amounts of secreted GFP, accounting for approximately 67.8% and 67.3% of the total protein in the medium, respectively. Intracellular GFPs accumulated stably, whereas secreted GFP underwent degradation during the prolonged period of cultivation, which can probably be ascribed to the activity of proteases in the medium (Figure S7). We also established that SP_GN_ and SP_Chi8_ were characterised by host‐ and method‐dependent secretion efficiencies. Moreover, these two SPs were demonstrated to be applicable for protein secretion in both *N. benthamiana* plants and suspension‐cultured cells, thereby yielding secreted proteins that can be protected from the degradative activity of proteases in the culture medium.

## 3 Discussion

The development and enhancement of plant expression vectors, along with the use of viral vectors and their integration with transient expression systems, have provided a basis for plants to serve as effective alternative production hosts for recombinant protein production. In addition, the FDA approval and marketing of ELELYSO (Taliglucerase alfa), the first plant‐made pharmaceutical (PMP) (Fox 2012; Mor 2015), and the successful clinical trials of ZMapp, an anti‐Ebola virus drug produced in *N. benthamiana* (McCarthy 2014; Olinger et al. 2012; Qiu et al. 2014), have accelerated research on recombinant protein production in plants. Accordingly, plants are increasingly recognised as attractive hosts for the production of pharmaceutical proteins. Recent research on plant protein production has primarily focused on upstream processes, such as vector enhancement, expression systems, post‐translational modifications, and control and/or regulation of plant growth conditions. Advances in these processes have contributed to increases in the biomass production of functional PMPs. However, although downstream processing accounts for the majority of the manufacturing costs (80% on average) (Raven et al. 2015; Schillberg et al. 2019; Wilken and Nikolov 2012), comparatively little attention has been paid to designing ideal downstream PMP production processes, including protein recovery, extraction, and purification steps, using techno‐economic analysis to assess the manufacturing process (McNulty et al. 2022). To facilitate and minimise downstream processing, several strategies have been examined, including fractionation, heat precipitation of host cell proteins, optimisation of extraction and clarification procedures, and aqueous two‐phase separation (Buyel and Fischer 2014; Wilken and Nikolov 2012). In addition, given that the secretion of recombinant proteins prevents intracellular proteolysis and facilitates subsequent recovery and purification processes (Karki et al. 2021), the establishment and development of effective secretion systems is desirable. Although the secretion of recombinant proteins into culture media is a versatile method for mammalian cells and microbes, such as *Escherichia coli* , yeasts (e.g., *Pichia pastoris*), and insects (e.g., *Spodoptera frugiperda* Sf9 cells), research on protein secretion in plants is still limited both *in planta* and in suspension‐cultured cells (Spiegel 2022). However, certain plant‐derived SPs can enhance the secretion of recombinant proteins (Jiang et al. 2020). Therefore, the use of novel plant‐derived SPs, ideally those native to the host plant, to facilitate recombinant protein secretion would be particularly desirable.

In this study, we isolated two novel SPs (SP_GN_ and SP_Chi8_) from the GN and Chi8 proteins induced in response to *Agrobacterium* infection. We demonstrated that these two SPs confer translocation/secretory capacities to different hosts and proteins, and can thus be used to facilitate protein secretion in both *N. benthamiana* plants and suspension‐cultured cells, provided that the secreted proteins can be protected from degradation by proteases in the culture medium. GN and Chi8 are members of the PR family of proteins that typically exhibit multiple functional properties in plants (Leubner‐Metzger and Meins 1999). Moreover, given their potential to degrade bacterial and fungal cell walls, PR proteins can elicit plant immune responses against pathogen infection‐induced stimuli (Jashni et al. 2015; Leubner‐Metzger and Meins 1999; Rivière et al. 2008). GN and Chi8 have been identified in *N. benthamiana* (Goulet et al. 2010), and the findings of recent studies have revealed that *Pseudomonas* infection promotes the production of PR proteins as antibacterial enzymes (Sueldo et al. 2023). Consequently, the expression of GN and Chi8 is ubiquitous as an early plant immune response to bacterial infection, which is consistent with their accumulation in the AWF following *Agrobacterium* infection (Van Loon et al. 2006). However, the SP regions of GN and Chi8 remain uncharacterised, and to date, there have been no reports on the contribution and use of these SPs for practical applications, such as facilitating the secretion of recombinant proteins. In this study, we demonstrated that native SP_GN_ and SP_Chi8_ can be used to facilitate protein secretion in both *N. benthamiana* plants and suspension‐cultured cells, with a higher efficiency than that of SP_Ext_, which is traditionally used as an SP. Notably, we established that GN and Chi8 were induced from 2 dpi onward and gradually accumulated in the AWF by 5 dpi. This indicates that both the SPs and promoters of GN and Chi8 are *Agrobacterium* infection‐inducible factors in *N. benthamiana*, and accordingly, these sequences could serve as *Agrobacterium* infection‐inducible elements for the production of recombinant proteins.

The apoplast, with its low protein diversity and overall protein content, provides an ideal environment for recombinant protein production compared with intracellular environments, as these properties are more conducive to purification. In addition, the process of protein secretion involves passage through the ER and Golgi apparatus, thereby ensuring post‐translational modifications, such as disulfide bond formation and *N*‐glycosylation, which are essential for protein activity. However, apoplasts are rich in proteases, including serine, cysteine, and aspartic proteases (Delannoy et al. 2008; Goulet et al. 2012), which can lead to challenges in stable protein accumulation and storage, which can hinder stable protein accumulation and storage (Lo Presti et al. 2015). Moreover, protease activity in the apoplast is exacerbated by bacterial infections, which further elicit protease production (Sueldo et al. 2023), notably in agroinfiltration‐based transient expression systems. In the present study, we found that PR proteins, such as GN and Chi8, were induced in response to agroinfiltration, whereas buffer infiltration alone had no significant effect on the protein composition of the apoplast (Figure 1a). This emphasises the importance of minimising protease activity triggered by bacterial infection for the stable production of recombinant proteins in plants. To address this issue and reduce proteolysis in planta and in suspension‐cultured cells, the efficacy of several strategies has been assessed, including the co‐expression of protease inhibitors (Jutras et al. 2019) and pH stabilisation in culture (Huang et al. 2009; Xu et al. 2011). In this study, GFP and GCase were successfully produced as active proteins in the AWF without significant degradation. However, other recombinant proteins are likely more susceptible to proteolytic degradation. Consequently, further studies using a broader range of proteins are required to assess the stability and yield of recombinant proteins in the apoplast. Furthermore, comprehensive proteomic analysis can provide valuable insights into protease activity and other factors that influence the production of recombinant proteins and their accumulation under agroinfiltration conditions.

Interestingly, we established that mammalian‐derived SPs can also mediate the translocation of target proteins to the apoplast (Figure 4), indicating that they are recognised by plant SRPs and cleaved by signal peptidases, similar to the corresponding processes in mammals. However, although mammalian SPs are functional in plants, their use for recombinant protein production may have detrimental side effects, such as tissue necrosis or, in severe cases, cell death. These issues can be minimised by using plant‐derived SPs or avoiding their use entirely (Gils et al. 2005; Wilbers et al. 2016). It is speculated that these adverse effects may stem from the accumulation of bioactive proteins to levels that become toxic to plants and/or could be associated with the stress caused by incorrect translocation/transport to a specific organelle as the final destination, rather than simply the protein burden. Although we detected no evidence of necrosis, even during the production of GFP, using viral vector systems that do not target specific organelles in plant tissues (Marillonnet et al. 2005, 2004), misfolded and/or immature recombinant proteins can induce tissue necrosis. This phenomenon can, however, be minimised by the co‐expression of molecular chaperones (Margolin et al. 2020), indicating that correct protein folding is essential for recombinant protein production in plants. Generally, apoplastic proteins have shorter amino acid sequences and fewer cysteine residues than intracellular proteins (Sperschneider et al. 2018). GFP is a suitable candidate for recombinant protein production in plants, particularly in the apoplast, as it has only two native cysteine residues (Cys48 and Cys70), a molecular weight of 26.8 kDa, and does not require intermolecular disulfide bonds (Dammeyer and Tinnefeld 2012). In contrast, GCase fails to meet these criteria, given that it has a molecular weight of 55.6 kDa without an SP and contains seven cysteine residues that form two disulfide bonds (Dvir et al. 2003). This indicates that target proteins, such as non‐functional enzymes in plants or plant enzymes with similar functions (e.g., glycoside hydrolases such as GCase), can be produced even if they are derived from mammalian sources. GCase is a member of the GH30 family of proteins, as classified in the CAZy database, whereas plants typically have numerous GH5 family proteins characterised by topologies and properties similar to those of GH30 (St John et al. 2010). Consequently, plants may successfully produce and accumulate active GCases in the apoplast (Figure 4f). To verify this hypothesis, it would be insightful to determine whether mammalian‐derived glycoside hydrolases in the same family as those in plants can be produced as secreted proteins.

We found that all assessed SPs facilitated the production of recombinant proteins alongside their secretion into the apoplast, whereas we detected virtually no GFP when this protein was fused to SP_BMP‐4_. Reference to the UniProt database revealed that SP_BMP‐4_ contains 19 N‐terminal amino acids (Table 1), with Gly at the putative C‐terminal end. Although SP_BMP‐4_ contains a central hydrophobic region that is presumed to form an α‐helix, it lacks other properties generally conserved in human SPs, such as an N‐terminal positively charged region (N‐region), C‐terminal uncharged region (C‐region), and a terminal Ala/Val‐X‐Ala motif (Liaci et al. 2021; Owji et al. 2018). The plant‐derived SPs identified to date tend to carry an Ala residue at the C‐terminus (Tables S2 and S3), which may be associated with misrecognition by SRPs and signal peptidases (SPases), resulting in the misfolding of the recombinant protein and subsequent degradation. Indeed, the findings of previous studies have indicated that low levels of expressed proteins result from the degradation of misfolded proteins (Gils et al. 2005). In contrast, although its efficiency was lower than that of plant‐derived SPs, SP_GCase_ facilitated the translocation of recombinant GCase into the apoplast (Figure 4). However, although SP_GCase_ meets the general structural criteria for an SP, its relatively long N‐region and weak hydrophobicity limit its efficacy as an SP, resulting in lower GCase secretion. Nevertheless, confirming these conjectures is challenging given the potential differences in the functions and recognition of plant SRPs and SPases compared with those in mammals. Consequently, the use of plant‐derived SPs is recommended for the efficient secretion of recombinant proteins into the apoplast and culture medium, particularly in suspension‐cultured cells. Although in this study we used two recombinant proteins for secretory production in plants, further studies are needed to fully assess the efficiency and efficacy of SP_GN_ and SP_Chi8_, as well as to confirm their utility in the secretory production of different proteins.

In this study, we demonstrated that the novel signal peptides, SP_GN_ and SP_Chi8_, are suitable for facilitating recombinant protein secretion in both intact *N. benthamiana* plants and suspension‐cultured cells. We established that exploiting endogenous signal peptides is a useful and effective strategy for enhancing the yield of secreted recombinant proteins in plant‐based expression systems. As an alternative to conventional recombinant protein production techniques, we accordingly propose a novel method for the production and purification of proteins of interest in plants.

## 4 Experimental Procedures

### 4.1 Plants and Growth Conditions

Seeds of wild‐type *N. benthamiana* were germinated in soil and grown at 25°C under 16 h light and 8 h dark conditions at 50% humidity. *N. benthamiana* suspension‐cultured cells were grown in Murashige and Skoog Basal Salt Mixture (Duchefa Biochemie, Haarlem, Netherlands), supplemented with 0.2 mg/L 2,4‐dichlorophenoxyacetic acid, under a rotary shaker set to 130 rpm at 25°C in dark conditions. About 10 mL of fresh cells were inoculated into 100 mL of fresh MS medium every 7 days for maintenance.

### 4.2 Plant Expression Vectors and *Agrobacterium* Strain

The GFP and GCase constructs, fused with SPs as shown in Table 1, as well as SP sequences derived from Ext, were prepared using PCR with KOD Plus Neo polymerase (TOYOBO, Osaka, Japan), templates (Limkul et al. 2016; Kajiura et al. 2010), and the primer set listed in Table S4. Primers containing signal sequences were designed to amplify GFP and GCase, each fused with the corresponding SP. The resultant fragments were introduced into pRI201‐AN (TaKaRa Bio, Shiga, Japan) by SLiCE (Motohashi 2017). The expression vector of P19, an RNA silencing suppressor from *Tomato bushy stunt virus*, was kindly provided by Prof. Atsushi Takeda, Ritsumeikan University, Japan. Each plant expression vector was independently introduced into *Agrobacterium tumefaciens* LBA4404 by electroporation.

### 4.3 *Agrobacterium*‐Mediated Transient Expression

Transient expression of target proteins in *N. benthamiana* was followed as previously described (Uthailak et al. 2021). Briefly, a single colony‐isolated *Agrobacterium* harbouring each plant expression vector was inoculated into 2 × YT liquid medium supplemented with the antibiotics kanamycin, rifampicin, and streptomycin and cultivated at 30°C overnight as the pre‐culture. The pre‐culture was inoculated into 2 × YT liquid medium (200 mL) supplemented with the same antibiotics and cultivated at 30°C for 24 h. Cells were collected by centrifugation at 4000 *g*. *Agrobacterium* containing different vectors (GFP, GCase, or P19) was resuspended and mixed at a 1:1 ratio in infiltration buffer (10 mM 2‐(*N*‐morpholino) ethanesulfonic acid, 10 mM MgSO_4_, pH 5.8) with OD_600_ of 0.20–0.25. *N. benthamiana* plants were infiltrated using vacuum infiltration as described in a previous report (Lombardi et al. 2012). Infiltrated plants were grown under normal growth conditions as mentioned above.

### 4.4 Sample Preparation of AWF and Intercellular Protein Extracts

Apoplast washing fluid (AWF) was prepared as previously reported (Jiang et al. 2020). Leaves at 5 days post‐infiltration (dpi) were submerged in AWF‐1 buffer (20 mM Tris–HCl (pH 8.0), 150 mM NaCl, 0.02% Silwet L‐77) in a steel dish and placed in a desiccator. Subsequently, the inside was under vacuum for 1 min. In the case of preparation for the GCase assay, AWF‐2 buffer (60 mM phosphate–citrate buffer pH 6.0, 4 mM β‐mercaptoethanol, 1.3 mM EDTA, 0.15% Triton X‐100, 0.125% taurocholic acid) was used instead of AWF‐1 buffer. The buffer‐infiltrated leaf was then placed on parafilm and rolled around the 1 mL tip. The leaf was put in a syringe, which in turn was put into a 50 mL conical tube and centrifuged at 1000 *g* for 10 min at room temperature. The eluate was further centrifuged at 15 000 *g* at 4°C for 5 min. The supernatant was collected as AWF. The residual leaf was used to extract the intracellular fraction and stored at −80°C until use.

Protein was extracted from suspension‐cultured cells as previously described with some modification (Sariyatun et al. 2021). Briefly, suspension‐cultured cells were collected by centrifugation at 15 000 *g* at 4°C for 5 min. Extraction buffer (100 mM Tris, 0.5 M NaCl, 0.5 M Arginine‐HCl, pH 7.0) was added at a ratio of 100 μL per 0.1 g of cells. Then, the cells were disrupted by sonication using the Handy Sonic UR‐20P (TOMY Seiko Co., Tokyo, Japan) six times for 30 s each on ice. The sample was again centrifuged at 15 000 *g* at 4°C for 5 min, and the supernatant was collected as the protein extract.

### 4.5 nanoLC‐MS/MS Analysis and *de Novo* Sequencing

The CBB‐stained bands corresponding to GN and Chi8, separated by SDS‐PAGE using a SuperSep Ace 10%–20% gradient gel (FUJIFILM Wako Pure Chemical Corporation, Osaka, Japan) and stained with CBB (CBB Stain One; nacalai tesque, Kyoto, Japan), were excised from the gel and cut into small pieces, then de‐stained completely with 50 mM NH_4_HCO_3_ in 50% acetonitrile and dehydrated twice using acetonitrile. The proteins in the gels were digested in‐gel as previously reported (Kajiura, Hiwasa‐Tanase, et al. 2022).

The trypsinised products were analysed using nanoLC‐MS/MS as previously reported (Kajiura, Tatematsu, et al. 2022). The MS data analysis, including peptide mapping, was performed using Data Analysis 4.0 software, BioTools, and SequenceEditor (Bruker Daltonics, Bremen, Germany). Fragmentation spectra were submitted to the MASCOT search engine (Matrix Science, http://www.matrixscience.com) against the SwissProt and NCBI protein databases for protein identification. Further protein identification was performed by conducting a BLAST search in the Sol Genomics Network database (https://solgenomics.net/).

### 4.6 Microscopic Analysis of GFP‐Expressing Leaves

The fluorescence microscopic analysis of the 7 dpi GFP‐expressing leaves was performed at 25°C using the Axio Observer Inverted microscope system (Carl Zeiss AG, Göttingen, Germany) equipped with a CFI Plan Apo20× lens and the ZEN Blue pro software (Carl Zeiss AG). For cell wall staining, calcofluor white (Sigma‐Aldrich, St Louis, MO) was diluted with 50% ethanol to 0.05 mg/mL. The fluorescence of GFP was excited using filters of 450–490 nm for excitation and 500–550 nm for emission, while that of calcofluor was excited using filters of 365 nm for excitation and 420–470 nm for emission. Image processing was performed using ZEISS ZEN 3.4 Pro image processing and Adobe Photoshop.

### 4.7 Measurement of GFP Intensity

GFP intensity in protein extract or AWF was measured using a FLUOstar Omega microplate reader (BGM Labtech, Offenburg, Germany) at λ_Ex_ = 485 nm and λ_Em_ = 520 nm. A standard curve was prepared using Recombinant GFP produced in *E. coli* (Sigma‐Aldrich). The intensity was calculated as ng GFP/μg of total soluble protein. For the measurement of total GFP intensity of *N. benthamiana* suspension‐cultured cells, 100 μL of cell suspension was directly measured, and the amount was calculated based on the intensity of GFP compared to recombinant GFP.

### 4.8 Quantitative Real‐Time PCR Analysis of Expressed *GFP*

Total RNA in transiently expressed *N. benthamiana* leaves was isolated using the QIAGEN RNeasy Plant Mini Kit (QIAGEN, Chatsworth, CA) and treated with DNase (NEB, Beverly, MA) to eliminate genomic DNA contamination. RNA aliquots (1.0 μg) were reverse‐transcribed using a Superscript VILO kit (Invitrogen, Carlsbad, CA). Quantitative real‐time PCR with the GoTaq qPCR Master Mix (Promega, Madison, WI) was performed using the prepared cDNA preparations as templates for the QuantStudio 3 Real‐Time PCR (Applied Biosystems, Foster City, CA). The primer sets used in this analysis are listed in Table S1. In every real‐time PCR run, *EF1α* was used as a control to normalise the amount of cDNA template. The relative expression levels were calculated using the transcript levels without any SP as the expression level of 1.0.

### 4.9 SDS‐PAGE and Western Blotting Analysis

All soluble protein samples were separated by SDS‐PAGE using a 12.5% or 7.5% acrylamide gel. The protein bands were visualised by CBB staining or Western blotting using anti‐GFP (Medical & Biological Laboratories Co., LTD., Aichi, Japan) or anti‐GCase (Sigma‐Aldrich) antibody as the primary antibody. Anti‐rabbit antibody conjugated to horseradish peroxidase (GE Healthcare, Tokyo, Japan) was used as the secondary antibody. The specific signals were visualised using Luminate Forte Western HRP Substrate (MILLIPORE, Billerica, MA) and detected by an iBright Imaging System (ThermoFisher Scientific, Tokyo, Japan).

### 4.10 GCase Activity Assay

GCase activity was measured as described previously (Uthailak et al. 2021). Briefly, crude leaf extract was co‐incubated with GCase assay buffer (60 mM phosphate–citrate buffer pH 6.0, 4 mM β‐mercaptoethanol, 1.3 mM EDTA, 0.15% Triton X‐100, 0.125% taurocholic acid) and 0.2 mM of 4‐methylumbelliferyl β‐D‐glucopyranoside (4‐MUG; FUJIFILM Wako Pure Chemical Corporation) at 37°C for 1 h in dark conditions. The enzymatic reaction was terminated by stop buffer (0.2 M glycine, 0.125 M Na_2_CO_3_, pH 10.8 adjusted by NaOH). The fluorescence of the reaction product, 4‐methylumbelliferone (4‐MU; FUJIFILM Wako Pure Chemical Corporation), was detected using an F‐7100 fluorescence spectrophotometer (Hitachi High‐Technologies, Tokyo, Japan) at *λ*_Ex_ = 365 nm and *λ*_Em_ = 460 nm. One enzyme unit was defined as the amount of enzyme used to release 1 nmol of 4‐MU/min, and each GCase activity was calculated as Units/mg of total soluble protein.

### 4.11 *N*‐Glycan Analysis of GCase Using nanoLC‐MS/MS

GCase transiently expressed in *N. benthamiana* leaves and secreted into AWF was excised. *N*‐Glycan analysis of the secreted GCase was performed as previously reported (Kajiura, Hiwasa‐Tanase, et al. 2022; Uthailak et al. 2021). The MS data analysis was performed using Data Analysis 4.0 software, BioTools, and SequenceEditor (Bruker Daltonics) as mentioned above.

### 4.12 Generation of GFP‐Expressing *N. benthamiana* Suspension‐Cultured Cells

Suspension‐cultured *N. benthamiana* cells were transformed by the *Agrobacterium*‐mediated transformation method with some modifications (Rempel and Nelson 1995). Three to four‐day‐old *N. benthamiana* suspension‐cultured cells were mixed with *Agrobacterium* harbouring each vector and kept at 25°C for 3 days under dark conditions. To remove *Agrobacterium*, infected cells were washed with MS medium containing 250 mg/L carbenicillin five times. The washed cells were further cultivated on a plate containing both 50 μg/L kanamycin and 250 μg/L carbenicillin for selection until calli were generated. The calli were increased to a sufficient volume and placed in 30 mL of MS medium containing kanamycin and carbenicillin. Cultures were incubated at 25°C, shaken at 130 rpm, and replanted every 7 days. In the growth assay, the cells were cultivated in 100 mL of culture.

### 4.13 Statistical Analysis

All statistical analyses were conducted using R (version 4.4.3). One‐way ANOVA followed by Tukey's post hoc test was performed to compare multiple groups. A *p*‐value of < 0.05 was considered statistically significant.

## Author Contributions

H.K. and K.F. designed the research. H.K. and K.Y. performed all experiments. H.K. and K.Y. performed all the statistical analyses. H.K., K.Y., and K.F. wrote the manuscript with support from R.M. All authors approved the final manuscript. K.F. supervised the research.

## Conflicts of Interest

The authors declare no conflicts of interest.

## Supporting information


**Figures S1–S7:** pbi70498‐sup‐0001‐FigureS1‐S7.pdf.

**Tables S1–S4:** pbi70498‐sup‐0002‐TablesS1‐S4.xlsx.

## Data Availability

The original dataset presented in this published article is included in the article and/or Supporting Information. The data that support the findings of this study are available on request from the corresponding author. The data are not publicly available due to privacy or ethical restrictions.
