# Supplementary material for: Protein‐Derived Signal Peptides Induced by Agrobacterium Infection Promote the Secretion of Recombinant Proteins in Nicotiana benthamiana
Source: Plant Biotechnol J. 2025 Dec 16;24(1):187–203. doi: 10.1111/pbi.70498 (PMC12854903; doi:10.1111/pbi.70498)
Supplement: Supplementary file 1 — Figures S1–S7: pbi70498‐sup‐0001‐FigureS1‐S7.pdf. [file PBI-24-187-s002.pdf]

Supplementary Materials

Protein-derived signal peptides induced by Agrobacterium infection promote secretion of recombinant proteins

Hiroyuki Kajiura, Kana Yamamoto, Ryo Misaki and Kazuhito Fujiyama

*** Correspondence:** Kazuhito Fujiyama: fujiyama@icb.osaka-u.ac.jp

endo-1,3-β-glucosidase (GN)

```
N. benthamiana     MALCIKN-GFLAALVLVGLLLCSIQMIGAQSIGVCYGKIANNLPSDQDVIKLYNANGIKR
N. tabacum         MTLCIKNGFLAAALVLVGLLICSIQMIGAQSIGVCYGKHANNLPSDQDVINLYNANGIRK
                   *:*****  : *********:***************** ***********:*******::

N. benthamiana     MRIYYPDTNVFNALKGSNIEIILDVPNQDLQSLTDVSRANGWVQDNIINHFPDVKFKYIA
N. tabacum         MRIYNPDTNVFNALRGSNIEIILDVPLQDLQSLTDPSRANGWVQDNIINHFPDVKFKYIA
                   **** *********:*********** ******** ************************

N. benthamiana     VGNEVSPGNNGQYAPFVAPAMQNVYNALAAAGLQDQIKVSTATYSGILENTYPPKDSIFR
N. tabacum         VGNEVSPGNNGQYAPFVAPAMQNVYNALAAAGLQDQIKVSTATYSGILANTYPPKDSIFR
                   ************************************************ ***********

N. benthamiana     EEFKSFTNPIIQFLARHNLPLLANIYPYFGHIYNSADVSLSYALFTQQGTNSAGYQNLFD
N. tabacum         GEFNSFINPIIQFLVQHNLPLLANVYPYFGHIFNTADVPLSYALFTQQEANPAGYQNLFD
                    **:** *******.:********:*******:*:***.********* :*.********

N. benthamiana     ALLDSMHFAVEKAGGQNVEIIVSESGWPSEGNSAATIENAQTYYTNLINHVKSGAGTPKK
N. tabacum         ALLDSMYFAVEKAGGQNVEIIVSESGWPSEGNSAATIENAQTYYENLINHVKSGAGTPKK
                   ******:************************************* ***************

N. benthamiana     PGKTIETYLFAIFDENNKEGDVTEKHFGLFSPDQRAKYQLNFN
N. tabacum         PGKAIETYLFAMFDENNKEGDITEKHFGLFSPDQRAKYQLNFN
                   ***:*******:*********:*********************
```

similarity: 90.9%

Chitinase 8 (Chi8)

```
N. benthamiana     MEFSGSPLVLFCCVFFLFLTGSLAQGIGSIVTSDLFNEMLKNRNDVRCPANGFYTYDAFI
N. tabacum         MEFSGSPMALFCCVFFLFLTGSLAQGIGSIVTSDLFNEMLKNRNDGRCPANGFYTYDAFI
                   *******:.************************************ **************

N. benthamiana     AAANSFPGFGTTGDDTARRKEIAAFFGQTSHETTGGSLSAEPFTGGYCFVRQNDQSERYY
N. tabacum         AAANSFPGFGTTGDDTARRKEIAAFFGQTSHETTGGSLSAEPFTGGYCFVRQNDQSDRYY
                   ********************************************************:***

N. benthamiana     GRGPIQLTNRNNYEKAGTAIQQDLVNNPDLVATDATISFKTAIWFWMTAQDNKPSSHDVI
N. tabacum         GRGPIQLTNRNNYEKAGTAIGQELVNNPDLVATDATISFKTAIWFWMTPQDNKPSSHDVI
                   ******************** *:*************************.***********

N. benthamiana     IGSWTPSAADQAANRVPGYGVITNIINGGIECGMGRNDAVEDRIGYYRRYCGMLNVAPGE
N. tabacum         IGRWTPSAADQAANRVPGYGVITNIINGGIECGIGRNDAVEDRIGYYRRYCGMLNVAPGE
                   ** ******************************:**************************

N. benthamiana     NLDCYNQRNFAQG
N. tabacum         NLDCYNQRNFGQG
                   **********.**
```

similarity: 96.0%

Figure S1. Alignments of amino acid sequences of GN and Chi8 from *Nicotiana tabacum* and *Nicotiana benthamiana*. The alignment was performed using CLUSTALW (http://align.genome.jp/). The peptide sequences detected in *de novo* sequencing are shown in red. Asterisks and colons indicate identical and similar amino acid sequences, respectively. Underlines represent the putative signal sequences.

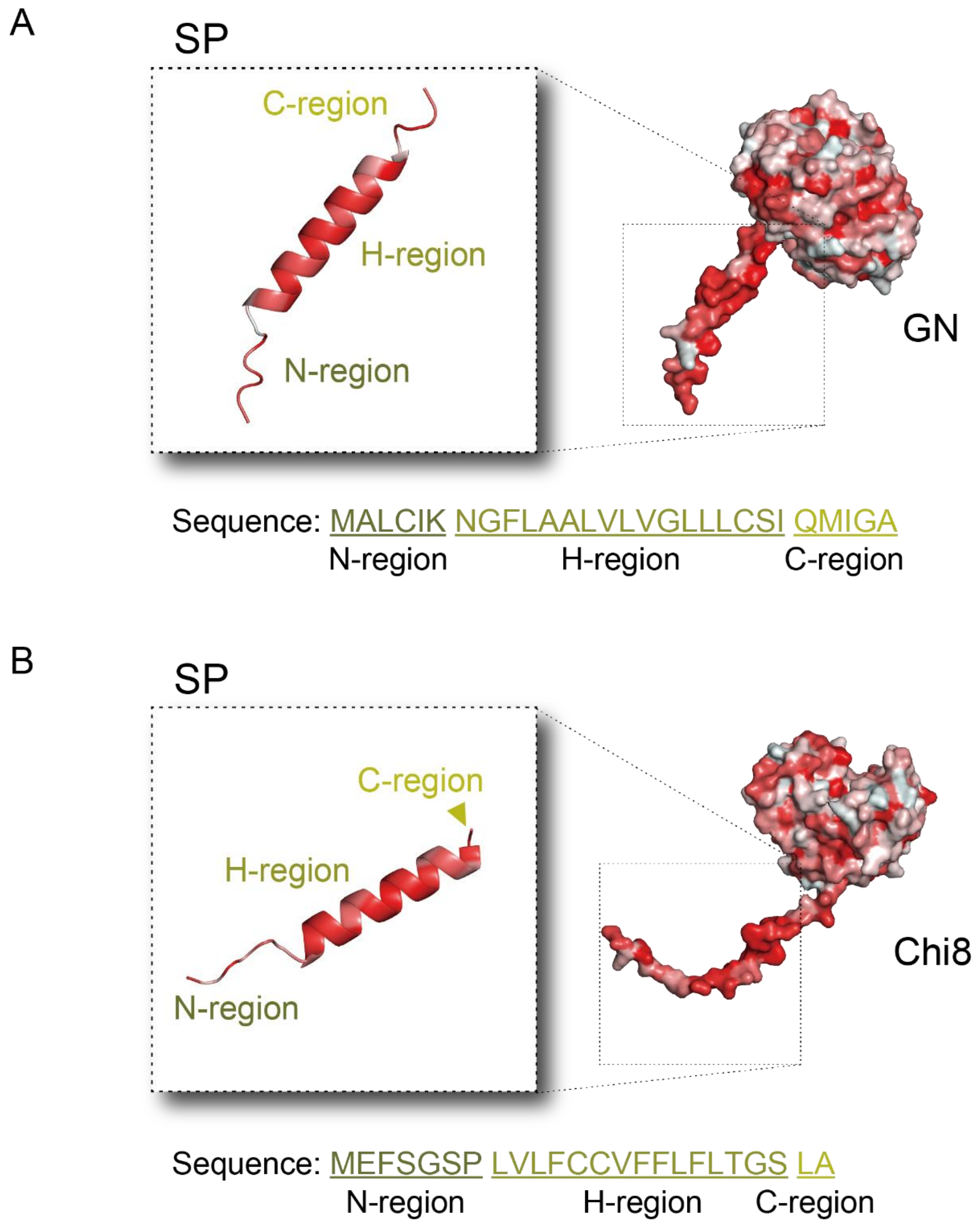

Figure S2. Homology modeling of GN and Chi8. Structural models of (A) GN and (B) Chi8 were constructed using Alphafold2. The N-terminal regions are shown in the cartoon structure, enlarged to show the putative N-, H-, and C-regions. Amino acids with high hydrophobicity are shown in red. The letters indicate the exact residues contributing to each region.

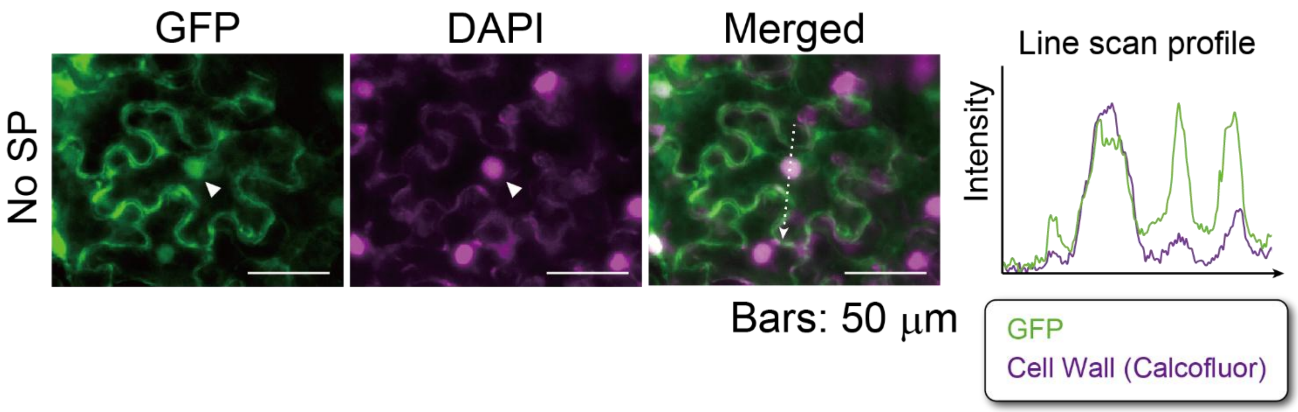

Figure S3. Microscopic analysis of 5-day-old *N. benthamiana* leaf epidermis cells. Dual-color imaging by fluorescent microscopy transiently GFP-expressing leaf. (Left) Light panel, GFP; middle panel, nucleus staining with DAPI; right panel merged images of these two fluorescence signals. White dotted arrows indicate the scanned line for line scan profile of each fluorescence intensity. White triangles indicate GFP signals in the nucleus. Bars: 50 μm. (Right) fluorescence intensity line scan profile generated along the white dotted arrow shown in merged figures. Green, the relative signal intensity of GFP; purple, the relative signal intensity of the nucleus stained with DAPI.

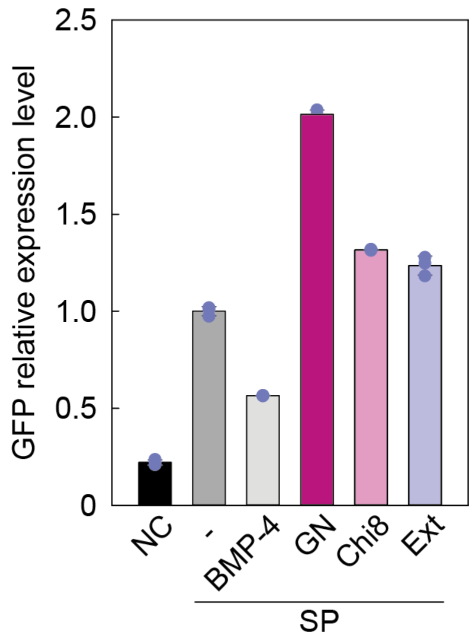

Figure S4. RT-PCR analysis of *GFP* expressed in transient expression system. Relative expression levels are shown. *EF1α* was used as a control. Error bars represent the standard deviation of the mean, and three replicates are shown as dots. The samples represent the following: NC (negative control, vector expression), - (GFP without signal peptide), BMP-4 (GFP fused with $SP_{BMP\text{-}4}$), GN (GFP fused with SP_{GN}), Chi8 (GFP fused with SP_{Chi8}), and Ext (GFP fused with SP_{Ext}).

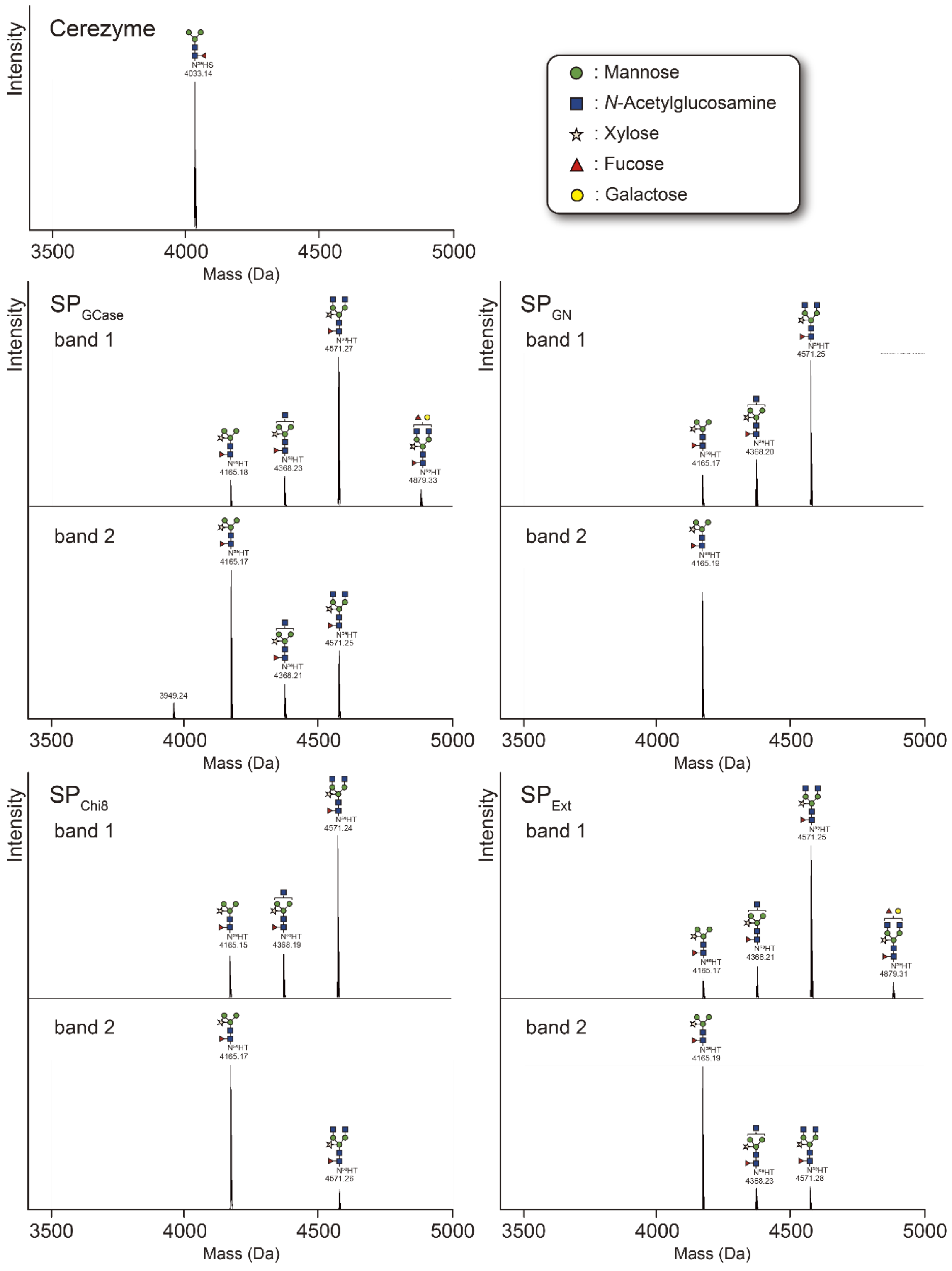

Figure S5. *N*-Glycosylation analysis of Asn59. All signals of *m/z* corresponding to *N*-glycopeptide and the *N*-glycan structures are shown. The *N*-glycopeptide was oxidized in two positions, presumably Met48 and Met53. The symbols used for *N*-glycan structures are shown in small window. The ratios of *N*-glycans are shown in Supplemental Table 2.

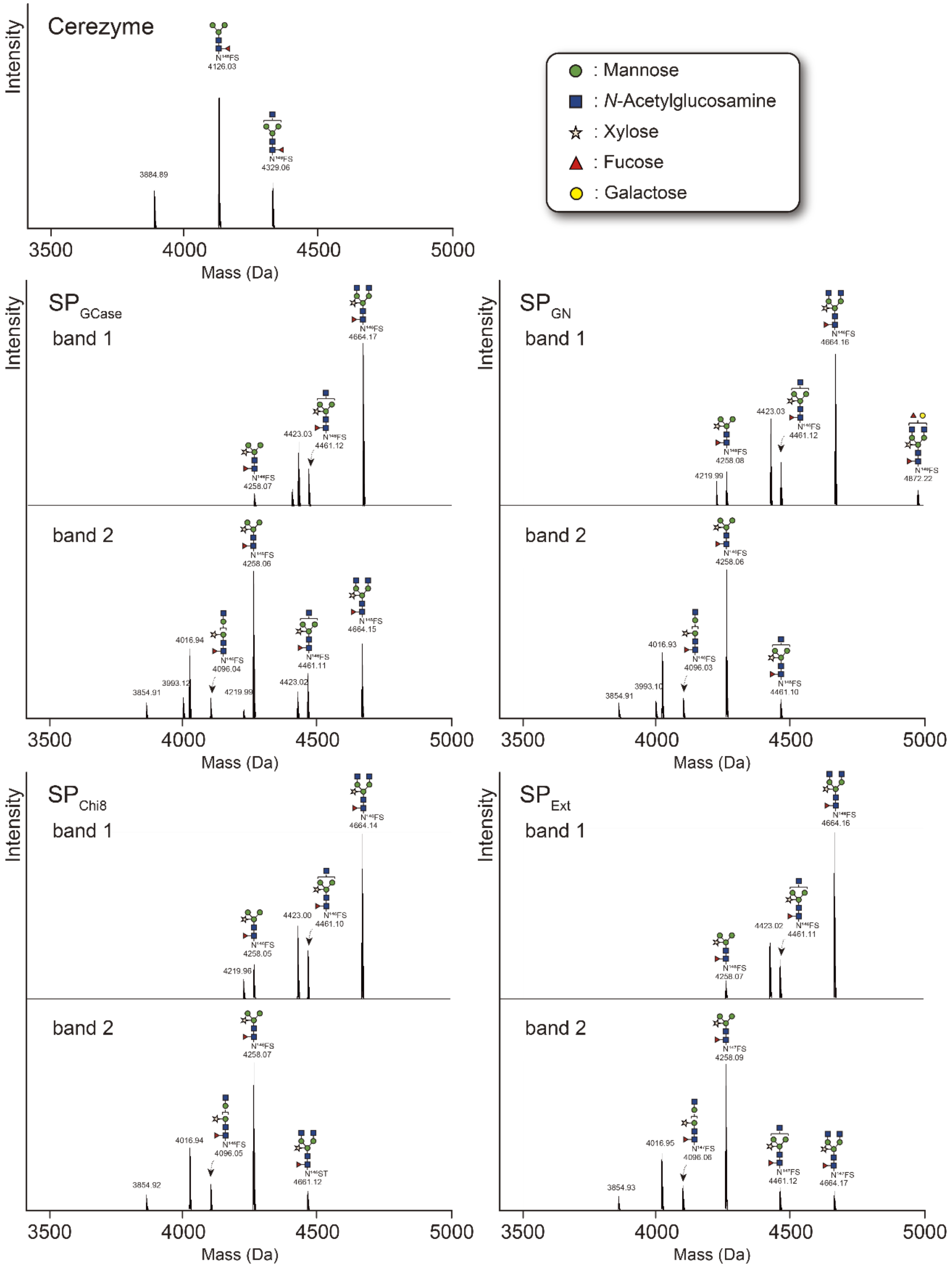

Figure S5, continued. ***N*-Glycosylation analysis of Asn146.**

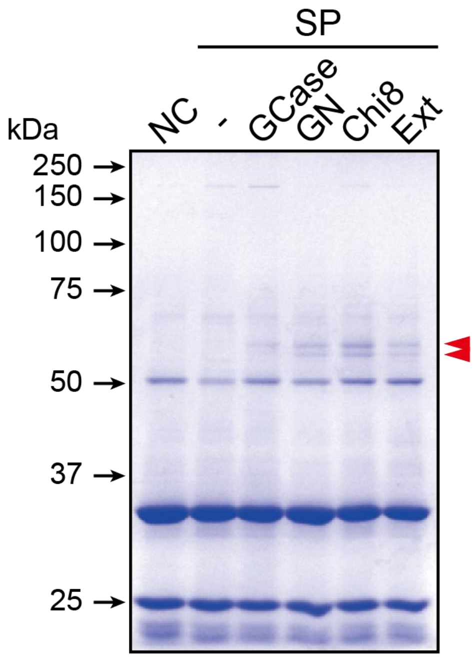

Figure S6. Transient production of GCase. The AWF proteins used in Figure 4 were separated by 12.5% acrylamide gel and stained by CBB. Red triangles indicate GCase produced in each transient expression.

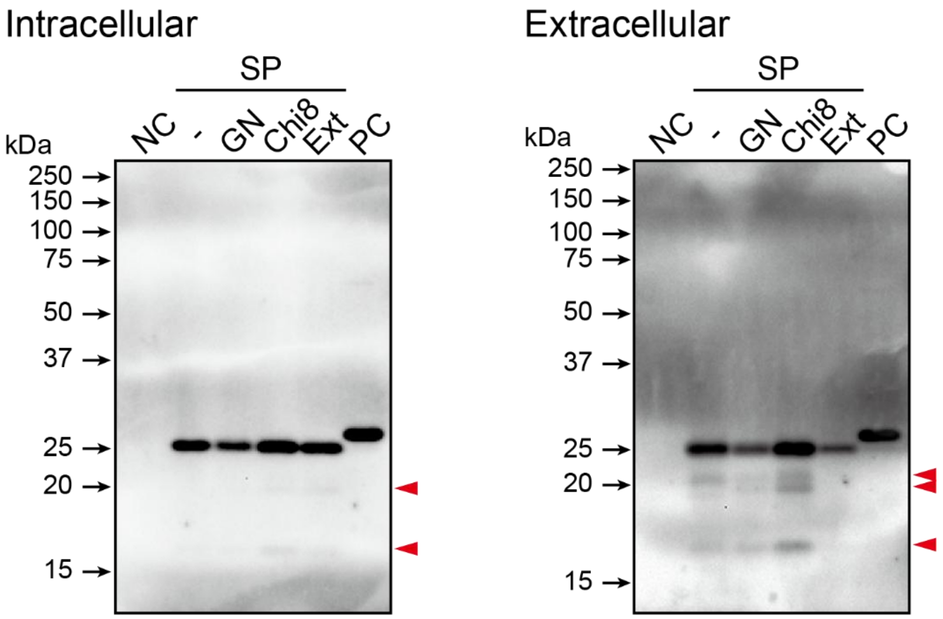

Figure S7. Degradation of secreted GFP. Long-time exposure of membrane used in Western blotting analysis of Figure 6A and B. Red triangles represent the degradation products of each GFP.
